# A LytM-Domain Factor, ActS, Functions in Two Distinctive Peptidoglycan Hydrolytic Pathways in *E. coli*

**DOI:** 10.3389/fmicb.2022.913949

**Published:** 2022-06-14

**Authors:** Pavan Kumar Chodisetti, Raj Bahadur, R. N. Amrutha, Manjula Reddy

**Affiliations:** Council of Scientific and Industrial Research-Centre for Cellular and Molecular Biology, Hyderabad, India

**Keywords:** bacteria, peptidoglycan, cell division, YgeR, MepS, ActS, AmiC

## Abstract

Bacterial cell wall contains peptidoglycan (PG) to protect the cells from turgor and environmental stress. PG consists of polymeric glycans cross-linked with each other by short peptide chains and forms an elastic mesh-like sacculus around the cytoplasmic membrane. Bacteria encode a plethora of PG hydrolytic enzymes of diverse specificity playing crucial roles in growth, division, or turnover of PG. In *Escherichia coli*, the cross-link-specific endopeptidases, MepS, -M, and -H, facilitate the enlargement of PG sacculus during cell elongation, whereas LytM-domain factors, EnvC and NlpD activate the division-specific amidases, AmiA, -B, and -C to facilitate the cell separation. In a screen to isolate additional factors involved in PG enlargement, we identified *actS* (encoding a LytM paralog, formerly *ygeR*) as its overexpression compensated the loss of elongation-specific endopeptidase, MepS. The overexpression of ActS resulted in the generation of partly denuded glycan strands in PG sacculi, indicating that ActS is either an amidase or an activator of amidase(s). The detailed genetic and biochemical analyses established that ActS is not a PG hydrolase, but an activator of the division-specific amidase, AmiC. However, interestingly, the suppression of the *mepS* growth defects by *actS* is not mediated through AmiC. The domain-deletion experiments confirmed the requirement of the N-terminal LysM domain of ActS for the activation of AmiC, but not for the alleviation of growth defects in *mepS* mutants, indicating that ActS performs two distinctive PG metabolic functions. Altogether our results suggest that in addition to activating the division-specific amidase, AmiC, ActS modulates yet another pathway that remains to be identified.

## Introduction

Bacterial cell wall contains a unique protective exoskeleton called peptidoglycan (PG) or murein. PG confers shape, provides structural integrity, and prevents osmotic lysis of bacteria. In Gram-negative bacteria, such as *Escherichia coli*, PG is located in the periplasmic space between the outer membrane (OM) and the inner membrane (IM). It is a single large molecule that forms a mesh-like sacculus around the cytoplasmic membrane. Structurally, it is made up of multiple linear glycan polymers interconnected by short cross-linked peptides (Höltje, 1998; Vollmer et al., 2008). Glycan chains consist of alternating disaccharide units of *N*-acetylglucosamine (GlcNAc) and *N*-acetylmuramic acid (MurNAc) residues linked together by a β-1,4-glycosidic bond. The lactoyl moiety of each MurNAc residue is covalently linked *via* an amide bond to a tetrapeptide chain, which typically comprises L-alanine (L-Ala)-γ-D-glutamic acid (D-Glu)-mesodiaminopimelic acid (mDAP)-D-Ala. In *E. coli*, approximately 30–40% of the peptide chains are cross-linked with each other either *via* D-Ala and mDAP residues (D-Ala^4^-mDAP^3^ or 4-3 cross-links) or two mDAP residues (mDAP^3^–mDAP^3^ or 3–3 cross-links). Of the total peptide cross-links, around 90–95% are of the 4-3 type, whereas 5–10% are of the 3-3 type (Vollmer et al., 2008).

Peptidoglycan precursor synthesis is initiated in the cytoplasm by the step-wise addition of amino acids to the nucleotide-activated sugar, UDP-MurNAc, to form UDP-MurNAc-pentapeptide (Bouhss et al., 2008). This is subsequently attached to the IM-bound lipid carrier, lipid-II. The monomeric lipid-II moieties are flipped across the IM to the periplasmic space, and subsequently polymerized to synthesize PG either for sidewall synthesis (during cell elongation) or septal wall synthesis at the mid-cell (during cell division). Two distinct multiprotein complexes, namely, elongasome and divisome, execute the side and septal wall syntheses, respectively (reviewed in Garde et al., 2021).

**FIGURE 1:**
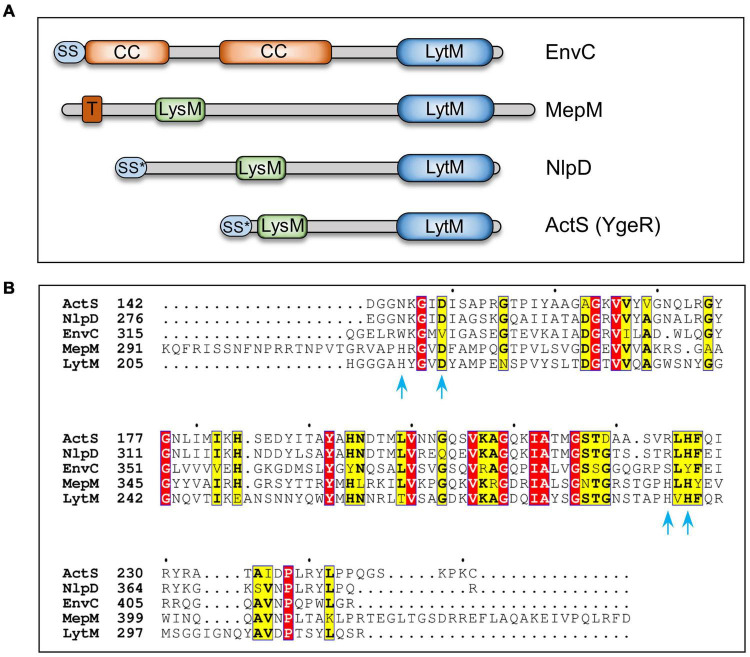
Domain organization and sequence alignment of LytM-domain factors. **(A)** Domain architecture of LytM factors (EnvC, MepM, NlpD, and ActS) of *Escherichia coli*. **(B)** Sequence alignment of C-terminal domains of LytM, EnvC, NlpD, MepM, and ActS using T-Coffee and ESPript programs (Di Tommaso et al., 2011, http://espript.ibcp.fr). LytM is a glycyl-glycine endopeptidase that cleaves peptidoglycan of *Staphylococcus aureus*. Amino acid residues of LytM involved in Zinc binding and catalysis (Firczuk et al., 2005) are indicated with blue arrows. Red and yellow boxes highlight the identities and similarities, respectively. LytM, lysostaphin/peptidase M23 domain; LysM, PG-binding domain; CC, coiled-coil domain; T, transmembrane domain; SS, signal sequence; SS*, lipoprotein signal sequence.

During bacterial cell elongation, the elongasomal complex synthesizes the side wall to facilitate PG expansion concomitantly with the growing cell volume. PG elongation requires cleavage of the cross-links in order to make space for the insertion of incoming nascent PG material and three cross-link specific *D,D*-endopeptidases- MepS, -M, and -H, are crucial for the growth of PG sacculus in *E. coli* (Singh et al., 2012). Among the elongation-specific PG hydrolases, MepS is highly expressed and stringently regulated at the level of post-translational stability (Singh et al., 2015). MepS and -H belong to the NlpC/P60 peptidase superfamily (Anantharaman and Aravind, 2003), whereas MepM belongs to the LytM or lysostaphin family of proteins (or M23 class of metallopeptidases). The other members of the LytM family are: EnvC, NlpD, and ActS (formerly known as YgeR; Figure 1). Unlike MepM, which is a cross-link specific endopeptidase, EnvC and NlpD do not have any enzymatic activity and are localized to the division site to facilitate cell–cell separation during cell division (Uehara et al., 2009).

The process of cell division is initiated by an essential cytoskeletal protein, FtsZ, which recruits several other divisomal proteins in a step-wise manner for the synthesis of septal PG in the mid cell (Levin and Janakiraman, 2021). Subsequently, EnvC and NlpD activate the division-specific amidases that specifically cleave the amide bonds between the MurNAc and L-Ala residues of the septal PG strands to enable the separation of the daughter cells. *E. coli* encodes three division-specific LytC-type *N*-acetylmuramyl-L-alanine amidases, AmiA, -B, and -C (Heidrich et al., 2001). EnvC stimulates the activity of AmiA and AmiB, whereas NlpD stimulates the activity of AmiC (Uehara et al., 2009; Peters et al., 2013). However, the function of ActS, an OM lipoprotein, has not been known until recently. While this work was in progress, two independent studies reported that ActS preferentially activates AmiC in OM stress (Gurnani Serrano et al., 2021) and AmiB in low pH conditions (Mueller et al., 2021).

Here, we identified *actS* as a multicopy suppressor of a mutant lacking *mepS*, the gene that encodes a major elongation-specific *D*,*D*-endopeptidase. Moreover, multiple copies of ActS in a WT strain led to the generation of partly denuded glycan strands in the PG sacculi suggesting that ActS is an amidase or an activator of amidase(s). However, ActS did not exhibit any detectable PG hydrolytic activity. Extensive PG analysis and microscopy of amidase mutants overexpressing *actS* indicated that ActS activates the division-specific amidase, AmiC, under physiological growth conditions. Interestingly, ActS suppression of *mepS* phenotype was not mediated through AmiC. Domain-deletion experiments further confirmed that the septal localization of ActS is crucial for AmiC activation but not for the alleviation of growth defects of *mepS* mutant, suggesting ActS directly or indirectly activates an alternate PG hydrolase that compensates the loss of MepS. We extensively tested the role of all known PG hydrolases in the suppression of the *mepS* phenotypes by ActS overexpression; however, none of these factors were found to be the candidates suggesting that ActS may either activate a yet unknown PG hydrolase or performs an alternate function independent of cell-wall hydrolysis.

## Materials and Methods

### Media, Bacterial Strains, and Plasmids

The LB medium has 0.5% yeast extract, 1% tryptone, and 1% NaCl (Miller, 1992). LBON is LB without NaCl. Nutrient Agar (NA) has 0.5% peptone and 0.3% beef extract. Minimal A media (Miller, 1992) was supplemented with 0.2% glucose and 0.4% casamino acids. Unless otherwise indicated, antibiotics were used in the following concentrations (μg/mL): ampicillin (Amp)—50, chloramphenicol (Cm)—30, and kanamycin (Kan)—50. The growth temperature was 37°C unless otherwise indicated. The bacterial strains and plasmids are listed in Supplementary Tables 2, 3.

### Molecular and Genetic Techniques

The experiments involving recombinant DNA and plasmid constructions were performed as per the standard methods. MG1655 genomic DNA was used as a template, and Phusion HF DNA polymerase (NEB) was used for PCR amplifications. Plasmid clones were always confirmed by sequence analysis. P1-phage-mediated transductions and transformations were performed using the standard methods (Miller, 1992). All strains are derivatives of MG1655 (Coli Genetic Stock Centre, Yale University) unless otherwise indicated. Deletion mutations are from the Keio mutant collection (NBRP, Japan) (Baba et al., 2006). Whenever required, the antibiotic resistance marker (Kan*^R^*) was flipped out using pCP20 plasmid encoding an Flp recombinase (Datsenko and Wanner, 2000).

### Screen for Identification of Multicopy Suppressors

A genetic screening was performed to identify the multicopy suppressors of Δ*mepS* mutant. A pool of ASKA plasmid library (a complete collection of plasmids carrying individual *E. coli* genes cloned downstream to an IPTG-inducible promoter) (Kitagawa et al., 2005) was transformed into a Δ*mepS* mutant strain, and the transformants were plated on NA plates supplemented with 10 μM IPTG. Δ*mepS* mutants do not grow at all on NA at 42°C, but grow poorly at 37°C. The selection for the suppressors was done at 37°C to allow the identification of weak suppressors. The plasmids that conferred moderate growth to Δ*mepS* strain were isolated and the ORFs conferring suppression were identified by sequencing.

### Viability Assays and Microscopy

The viability of the strains was checked by growing the strains overnight in permissive conditions, serially diluting (10^–2^, 10^–4^, 10^–5^, and 10^–6^) the strains, and placing 5 μL aliquots of each dilution onto the required plates followed by incubation at 37°C for 18–24 h. For microscopy, the cultures were immobilized on a thin 1% agarose pad and visualized using Axioimager microscope by DIC (Nomarski Optics).

### Preparation of Peptidoglycan Sacculi

The isolation of PG was done as described earlier (Glauner et al., 1988). The cells (from 1,000 ml culture) grown to OD_600_ of 1.0 were harvested by centrifugation at 10,000 × *g* for 10 min at 4°C. Cell pellet was resuspended in 6 ml of ice-cold deionized water and added drop wise into 6 ml of boiling 8% SDS with vigorous stirring followed by boiling for another 45 min. The mixture was incubated overnight at room temperature. The PG pellet was washed thoroughly with deionized water to remove SDS by high-speed centrifugation (2,00,000 × *g* for 60 min). High-molecular weight glycogen and covalently bound lipoprotein, Lpp, were removed by treating with α-amylase (100 μg/ml in 10 mM Tris–HCl, pH 7.0, 2 h at 37°C) and pre-digested pronase (200 μg/ml, 90 min at 60°C). Enzymes were inactivated by boiling with an equal volume of 8% SDS for 15 min. Pure PG sacculi were obtained by ultracentrifugation and washed several times with water till SDS was completely removed. The final pellet was resuspended in 0.5 ml of 25 mM Tris–HCl (pH 8.0) and stored at −30°C.

### Analysis of Peptidoglycan Sacculi

Peptidoglycan analysis was done as described earlier (Glauner et al., 1988). Essentially, the sacculi were digested with 10 U mutanolysin (Sigma-Aldrich, St. Louis, MO, United States) at 37°C in 25 mM Tris–HCl (pH 8.0) for 16 h. Mutanolysin hydrolyzes β-1→4 glycosidic bond between MurNAc and GlcNAc residues in PG to form soluble muropeptide fragments. After centrifugation at 30,000 × *g* for 15 min, soluble muropeptide fragments in supernatant fraction were collected and reduced with 1 mg of sodium borohydride in 50 mM sodium borate buffer (pH 9.0) for 30 min and excess borohydride was destroyed by adding 20% phosphoric acid. pH was adjusted to 3–4 and the samples were loaded onto a reverse-phase C18 column (Zorbax 300 SB; 250 mm × 4.6 mm, 5 mm) connected to Agilent technologies RRLC 1200 system. Column temperature was 55°C and binding was done at a flow rate of 0.5 ml/min with 1% acetonitrile in water containing 0.1% trifluoroacetic acid (TFA) for 10 min. Muropeptides were eluted in a gradient of 1–10% acetonitrile containing 0.1% TFA at a flow rate of 0.5 ml/min for the next 60 min (using RRLC online software called Chemstation). The absorbance of muropeptides was detected at 205 nm.

### Mass Spectrometry Analysis of Muropeptides

The muropeptide fractions collected during high-pressure liquid chromatography (HPLC) were dried and reconstituted in 5% acetonitrile with 0.1% formic acid and loaded onto a reverse-phase PepMap™ RSLC – C18 column (3 μm, 100 Å,75 μm × 15 cm) connected to Q-Exactive™ HF Hybrid Quadrupole-Orbitrap™ Mass Spectrometer (Thermo Fisher Scientific, Waltham, MA, United States). The peaks were analyzed using mass spectrometry (MS) and the structures were decoded based on molecular mass of the fragments.

### Protein Purification

ActS-encoding plasmid, pET21b-ActS^27–251^, was transformed into BL21 λDE3 strain and a single transformant was inoculated into 10 ml LB broth with ampicillin and grown overnight. The culture was diluted 1:100 into a fresh pre-warmed LB broth with Amp, and was allowed to grow until OD_600_ of ∼0.6 was reached before being induced by the addition of 50 μM IPTG and grown further for 2 h at 37°C. The cells were recovered by centrifugation and the pellet was stored at −80°C until further use. The cell pellet was resuspended in 20 ml lysis buffer (50 mM Tris, 300 mM NaCl, 10 mM imidazole, pH 8.0) and was lysed by sonication (20% Amplitude; 10 s on–off). The cell debris was removed by centrifugation at 30,000 × *g* for 30 min at 4°C. The supernatant was mixed with 1 ml Ni^2+^-NTA agarose (Qiagen, Hilden, Germany) and mixed at 4°C for 1 h. This mixture was loaded into empty plastic column (Bio-Rad, Hercules, CA, United States) and washed with 30 ml wash buffer-I (50 mM Tris, 300 mM NaCl, 20 mM imidazole, pH 8.0), 10 ml of wash buffer-II (50 mM Tris, 300 mM NaCl, 50 mM imidazole, pH 8.0), 10 ml of elution buffer-I (50 mM Tris, 300 mM NaCl, 100 mM imidazole pH 8.0), and 5 ml of elution buffer-II (50 mM Tris, 300 mM NaCl, 200 mM imidazole pH 8.0). Purified protein was pooled and concentrated to 2.5 ml using a 3 kDa cut-off centrifugal membrane filter (Millipore). This eluate was loaded onto a buffer exchange PD10 column (Amersham) and retained proteins were eluted in 3.5 ml storage buffer (100 mM Tris, 200 mM NaCl, and 2 mM DTT). The protein was concentrated to 250 μL by a 3 kDa cut-off centrifugal membrane filter before being mixed with an equal volume of 100% glycerol and stored at −30°C.

ActS^130–251^ protein was purified using the plasmid pET21b-ActS^130–251^, as described earlier, except that the induction for protein overexpression was for 2.5 h at 30°C.

### Purification of CwlO

The C-terminal region of CwlO of *Bacillus subtilis* was overexpressed and purified to homogeneity using the plasmid, pET21b-CwlO^340–473^, as described earlier, except that the induction for protein overexpression was done with 0.1 mM IPTG for 2 h at 30°C.

### Zymogram Assay

Zymography of the proteins was performed as described earlier (Bernhardt and de Boer, 2004). Purified proteins were electrophoresed on two 12% SDS-gels, in which one gel was impregnated with *Micrococcus lysodeikticus* cells. After electrophoresis, this gel was incubated overnight in a renaturation buffer (25 mM Tris–Cl, pH 8.0, and 1% Triton X-100) for refolding the proteins. The gel was stained with methylene blue (0.1% methylene blue in 0.01% KOH) to visualize the cleared hydrolytic zones in blue background. The other gel stained with Coomassie brilliant blue was used as control.

### Determination of Enzyme Activity

The total soluble muropeptides or intact PG sacculi were incubated with purified proteins, ActS^FL^, ActS^LytM^, or CwlO, at 30°C for 20 h. The samples were heat-inactivated (100°C, 5 min) and analyzed by reverse-phase-HPLC (RP-HPLC), as described earlier.

### Western Blot Analysis

The samples were boiled with Laemmli loading dye and the proteins were separated by SDS-PAGE. Primary α-His antibodies were used at 1:3,000 dilution. Secondary anti-mouse-HRP conjugate antibodies were used at a dilution of 1:10,000 and blots were developed with ECL chemiluminescent detection kit (GE Biosciences, Chicago, IL, United States).

## Results

### Identification of ActS as a Multicopy Suppressor of *mepS*

**FIGURE 2:**
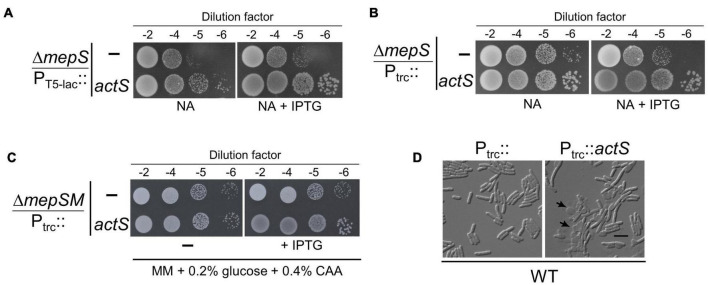
Phenotypes caused by ActS overexpression. **(A)** Growth of Δ*mepS* mutant carrying the ASKA vector (P_T5–lac_::) or vector encoding *actS* (P_T5–lac_::*actS*) on NA plates supplemented with 10 μM isopropyl-thiogalactopyranoside (IPTG) at 37°C. **(B)** Growth of Δ*mepS* mutant carrying the empty vector (P_trc_::) or vector encoding *actS* (P_trc_::*actS*) on NA plates supplemented with 20 μM IPTG at 37°C. **(C)** Δ*mepS* Δ*mepM* double mutant harboring either pTRC99a (vector) or vector encoding *actS* (P_trc_::*actS*) were grown in minimal media (MM) and tested for viability on MM plates supplemented with 0.2% glucose, 0.4% casamino acids (CAA), and 20 μM IPTG at 37°C. **(D)** WT cells carrying the vector (P_trc_::) or vector encoding *actS* (P_trc_::*actS*) were grown in LB supplemented with 50 μM IPTG at 37°C, harvested at 0.5 OD, and subjected to microscopy (DIC) as described in the “Materials and Methods” section. Arrows indicate cell lysis, and the scale bar represents 5 μm.

MepS is a major elongation-specific PG hydrolase that exhibits D,D-endopeptidase activity on 4-3 (D-ala^4^-mDAP^3^) cross-links to make space for the insertion of new material during the expansion of PG (Singh et al., 2012). A mutant of *E. coli* lacking *mepS* does not grow on low-osmolar media such as NA at high temperatures (Hara et al., 1996). To identify the additional factors involved in PG enlargement, we took advantage of the NA-sensitivity phenotype of the *mepS* deletion mutant and screened the ASKA plasmid library [a complete collection of plasmids carrying individual *E. coli* genes cloned downstream to an IPTG inducible promoter (P_*T*5–lac_::)] (Kitagawa et al., 2005). This plasmid library was pooled and transformed into Δ*mepS* cells to obtain suppressors on NA plates (as described in the “Materials and Methods” section). Of the several suppressors obtained, we observed that a plasmid harboring an ORF, *ygeR* (*actS*) [pCA24N-*actS*] conferred moderate growth advantage to Δ*mepS* mutant (Figure 2A).

To further confirm the above observation, full-length *actS* was cloned under IPTG-inducible *trc* promoter (P_trc_::) in a pTRC99a vector and introduced into Δ*mepS* mutant. Viability assays indicated that overexpression of *actS* conferred moderate growth to Δ*mepS* mutant on NA similar to that of pCA24N-*actS* (Figure 2B). Next, to check whether overexpression of *actS* can compensate for the loss of both *mepS* and *mepM*, we introduced pTRC99a-*actS* into a Δ*mepS* Δ*mepM* double mutant. It is known earlier that Δ*mepS* Δ*mepM* double mutants do not grow on rich media but grow on defined media (Minimal medium, MM) (Singh et al., 2012). Figure 2C shows that overexpression of *actS* weakly suppresses the growth defects of Δ*mepSM* double mutant. However, *actS* deletion did not confer any noticeable growth defect singly or when introduced into either Δ*mepS* or Δ*mepSM* mutants (data not shown). Interestingly, overexpression of *actS* in WT caused extensive cell lysis and microscopic (DIC) examination revealed misshapen cells with cellular contents oozing out into the medium (Figure 2D and Supplementary Figures 1A,B). All these observations collectively implied that ActS is either a PG hydrolase or activates an alternate hydrolase.

### ActS Overexpression Generates Glycan Chains Lacking Stem Peptides

**FIGURE 3:**
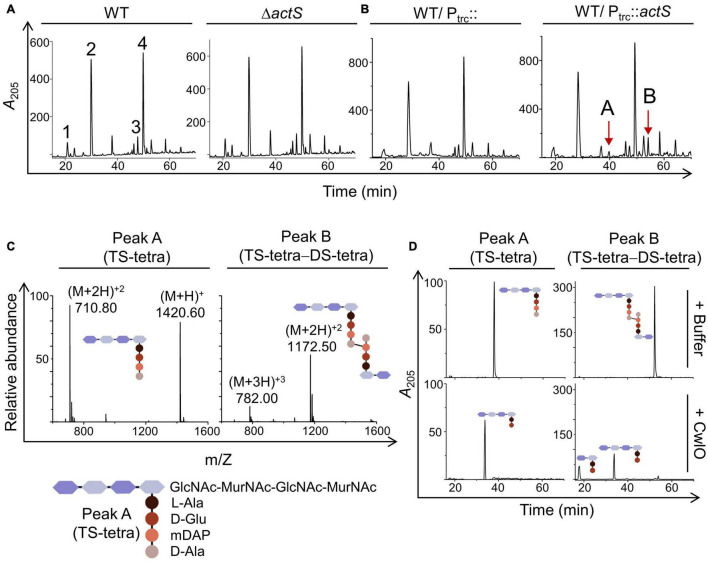
Effect of *actS* on the composition of PG. **(A)** HPLC chromatograms depicting the PG composition of WT and Δ*actS* mutant. Peak 1: disaccharide tripeptide (tri), peak 2: disaccharide tetrapeptide (tetra), peak 3: tri-tetra (3-3), peak 4 - tetra-tetra (4-3). **(B)** HPLC chromatograms depicting the PG composition of cells overexpressing ActS. The additional muropeptide peaks are represented as peaks A and B. **(C)** Mass spectra of peaks A and B with molecular mass of 1,420 Da [(M + 2H)^+2^ of 710.80 Da] and 2,346 Da [(M + 2H)^+2^ of 1,172.50 Da], respectively. Peak A is tetrasaccharide tetrapeptide (TS-tetra), whereas peak B is tetrasaccharide tetrapeptide cross-linked to disaccharide tetrapeptide (TS-tetra-DS-tetra). Structures are depicted in the inset. **(D)** Purified TS-tetra and TS-tetra-DS-tetra were incubated either with buffer or purified CwlO (5 μM) for 20 h at 30°C and separated by RP-HPLC.

To further investigate the role of ActS, we examined the PG composition of cells lacking *actS*. PG sacculi from both WT and Δ*actS* strains were purified and subjected to muramidase (mutanolysin) digestion to obtain soluble muropeptides. These muropeptide fragments were fractionated by RP-HPLC. Figure 3A shows that the PG sacculi isolated from Δ*actS* cells did not have any noticeable changes compared to that of WT cells. Next, we examined the PG composition of WT cells overexpressing *actS*. Interestingly, HPLC chromatograms revealed the presence of two additional peaks with retention times of 43 min (peak A) and 54 min (peak B) (Figure 3B). MS analysis indicated peak A to be a monomer of tetrasaccharide tetrapeptide (abbreviated TS-tetra) and peak B as a heterodimer of tetrasaccharide tetrapeptide cross-linked to a disaccharide tetrapeptide (abbreviated TS-tetra-DS-tetra) with a molecular mass of 1,420 and 2,346 Da, respectively (Figure 3C).

The molecular identities of peak A (TS-tetra) and peak B (TS-tetra-DS-tetra) were verified after subjecting them to enzymatic digestion and further analysis of the resultant fragments. For this purpose, a *D,L*-endopeptidase, CwlO, from *Bacillus subtilis* that cleaves between D-Glu and mDAP within the stem peptide of PG was used (Yamaguchi et al., 2004). A hexahistidine-tagged truncated version of CwlO (*cwlO*^340–473^-His_6_) was cloned, purified, and shown to be active against the purified fraction of TS-tetra and TS-tetra-DS-tetra (Figure 3D). The molecular mass of the resultant peaks after CwlO cleavage was determined by MS analysis, which further validated the identity of peaks A and B to be TS-tetra and TS-tetra-DS-tetra, respectively. The presence of these non-canonical muropeptides allowed us to infer that the PG sacculi of *actS*-overexpressed cells contain partly denuded glycan chains which can only be produced by the activity of amidases. The above results suggested that ActS either can be an amidase or may activate other amidase(s).

### ActS Has No Detectable Peptidoglycan Hydrolytic Activity

**FIGURE 4:**
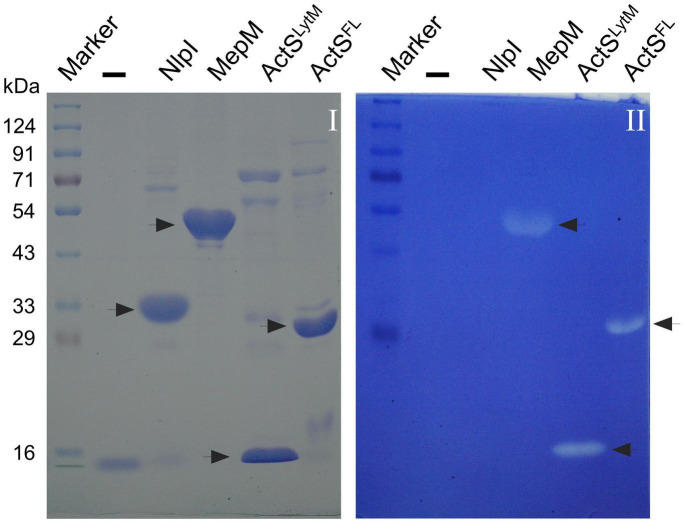
Zymogram assay showing the activity of ActS. Indicated proteins were electrophoresed and the zymogram assay was done as described in the “Materials and Methods” section. Gel-I is stained with Coomassie blue and gel-II containing the *M. lysodeikticus* cells is stained with methylene blue. Purified ActS^FL^ (lane 6) and ActS^LytM^ (lane 5) exhibit zone of clearance. NlpI (lane 3), a lipoprotein serves as a negative control whereas MepM (lane 4) is a positive control.

To examine whether ActS has any activity on PG sacculi or soluble muropeptides, we cloned, overexpressed, and purified signal-less, hexahistidine-tagged ActS derivative [ActS^27–251^-His_6_] by Ni-NTA affinity chromatography. ActS was subjected to zymogram analysis in which a zone of clearance was observed indicating that ActS may either possess PG binding or cleavage activity (Figure 4). However, incubation of soluble muropeptides or intact PG sacculi with ActS did not show any cleavage activity under several reaction conditions (data not shown). To test whether the LysM domain is interfering with the biochemical activity, we made a construct overexpressing only the LytM domain of ActS and purified the truncated variant of ActS, ActS^130–251^-His_6_ (ActS*^LytM^*). ActS*^LytM^* also displayed a zone of clearance in the zymogram assay (Figure 4) but did not exhibit any cleavage activity on soluble muropeptides or intact PG sacculi (data not shown). These results are consistent with the observation of ActS lacking two of the essential catalytic residues in the LytM domain (Figure 1; Uehara et al., 2010) and allowed us to conclude that ActS does not possess any PG hydrolytic activity.

### ActS Activates the Division-Specific Amidase, AmiC

**FIGURE 5:**
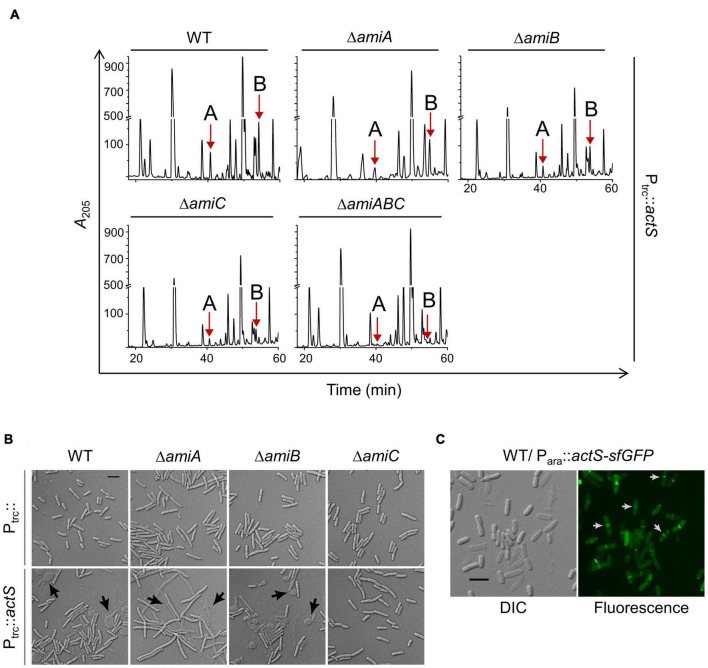
Effect of ActS overexpression on WT and division-specific amidase mutants. **(A)** HPLC chromatograms depicting the PG composition of WT, Δ*amiA*, Δ*amiB*, Δ*amiC*, or Δ*amiABC* mutants with overexpressed *actS* (P_trc_::*actS*). Strains were grown to an OD_600_ of ∼1 in LB containing 50 μM IPTG followed by isolation and analysis of PG sacculi. **(B)** The strains were grown overnight and diluted 1:100 into fresh LB broth supplemented with 50 μM IPTG and grown till OD_600_ of 0.5. Cells were collected and subjected to microscopy as described the “Materials and Methods” section. Arrows indicate cell lysis, and the scale bar represents 5 μm. **(C)** Localization of ActS-sfGFP was done in WT cells carrying pBAD18*actS*-sfGFP. Cells were grown with 0.2% arabinose and harvested at ∼0.4 OD and visualized by microscopy as described in the Materials and Methods. Arrows (in white) indicate ActS localization in cells with deep constriction. The scale bar represents 5 μm.

As ActS did not show amidase activity, we presumed that it may activate any of the division-specific amidases (AmiA, -B, and -C). To examine this possibility, we introduced pTRC99a-*actS* plasmid into single amidase deletion mutants lacking either *amiA*, -*B*, or -*C* and analyzed the composition of PG sacculi of these strains. The HPLC chromatograms clearly showed that the peaks corresponding to TS-tetra and TS-tetra-DS-tetra muropeptides were significantly lower in Δ*amiC* mutant compared to that of Δ*amiA* or Δ*amiB* mutants (Figure 5A and Supplementary Table 1). Additionally, these non-canonical muropeptides were completely absent in a Δ*amiABC* triple mutant overexpressing *actS* (Figure 5A). As the overproduction of ActS elicited lysis in WT cells (Figure 2D), we examined whether the lysis is mediated through any of these amidases. The viability assays indicated that the overexpression of *actS* confers sickness to AmiA and AmiB deletion mutants but not to AmiC deletion mutants (Supplementary Figure 1A). Accordingly, microscopic observations confirmed the lysis of WT, AmiA, and AmiB mutants but not that of AmiC mutant upon ActS overexpression (Figure 5B). Furthermore, cell morphology of various double amidase mutants (Δ*amiAB*, Δ*amiBC*, or Δ*amiAC*) overexpressing *actS* confirmed that the lysis is mostly mediated by AmiC (Supplementary Figure 1B). Altogether, these observations suggest that ActS predominantly activates the amidase, AmiC.

Since AmiC is a part of the divisomal complex that is recruited to the septum to facilitate cell–cell separation, ActS is also likely to be localized to the cell septa. Therefore, to examine the localization of ActS, we cloned it in the pBAD18-sfGFP vector under an arabinose-inducible promoter (P_*ara*_::*actS-sfgfp*). Figure 5C shows septal localization of ActS-sfGFP mostly in cells having a deep and visible constriction at the mid-cell indicating ActS is a late-division protein. It has also been shown earlier that ActS (YgeR)–mCherry fusion exhibits weak septal localization (Uehara et al., 2009).

### Activation of AmiC by ActS Is Not the Basis for Suppression of Δ*mepS* Growth Defect

**FIGURE 6:**
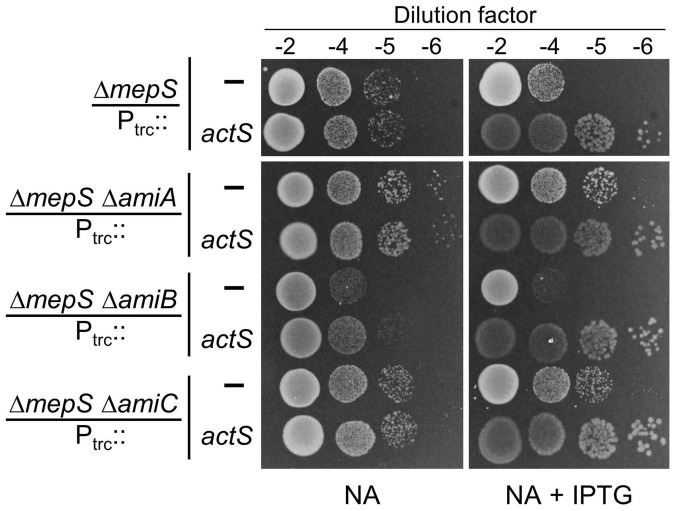
Alleviation of Δ*mepS* growth defects by ActS is not mediated by AmiC. Indicated strains carrying empty vector (P_trc_::) or vector encoding *actS* (P_trc_::*actS*) were subjected to viability assay on NA plates with (20 μM) or without IPTG. For reasons not clear, loss of AmiA or AmiC conferred a subtle growth advantage to Δ*mepS* mutant.

As the above results indicated the activation of AmiC by ActS, next, we examined whether suppression of Δ*mepS* growth defects by overexpression of ActS is mediated through AmiC. For this purpose, we introduced *amiA*, -*B*, or -*C* single gene deletions into the Δ*mepS* mutant (to construct Δ*mepS* Δ*amiA*, Δ*mepS* Δ*amiB*, and Δ*mepS* Δ*amiC* double mutants) and examined the effect of *actS* overexpression on their growth. Interestingly, multiple copies of *actS* were able to suppress the NA-sensitivity of *mepS* deletion mutant lacking any of the amidase genes similar to that of *mepS* single mutant demonstrating that the suppression is not mediated through activation of any of these amidases (Figure 6). Moreover, plasmids overexpressing AmiA, -B, or -C did not rescue the Δ*mepS* mutant growth phenotypes corroborating the above observations (Supplementary Figure 2). These results ruled out the role of AmiA, -B, and -C in the suppression of Δ*mepS* mutant, implicating the existence of an alternative pathway as the basis of its growth rescue.

**FIGURE 7:**
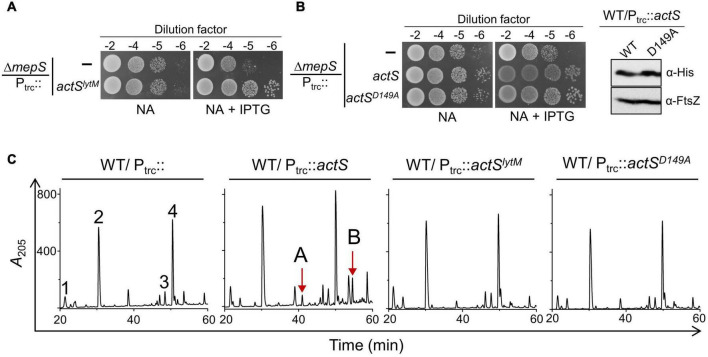
Overexpression phenotypes of ActS variants. **(A)** Viability assay indicating the suppression of Δ*mepS* phenotype by ActS^LytM^. **(B)** Viability assay indicating the suppression of Δ*mepS* phenotype by ActS^FL^-His and ActS^D149A^-His (B.1). Western blot showing the expression of ActS^WT^-His and ActS^D149A^-His induced with 20 μM IPTG (B.2). **(C)** HPLC chromatograms depicting the composition of PG of cells carrying either *actS* (P_trc_::*actS*), *actS^LytM^* (P_*trc*_::*actS*^LytM^) or *actS^D149A^* (P_trc_::*actS*^*D*149*A*^). Strains were grown to an OD_600_ of ∼1 in LB containing 500 μM IPTG followed by isolation and analysis of PG sacculi.

To examine whether the septal localization of ActS is important for the suppression of Δ*mepS* phenotype, we deleted the LysM domain of ActS, which is responsible for the septal recruitment (Poggio et al., 2010; Tsang et al., 2017) to construct a truncated version containing only the LytM domain (P_trc_::*actS*^Δ^*^lysM^* or *actS^lytM^*). Multiple copies of *actS^lytM^* also suppressed Δ*mepS* growth defects like that of the WT *actS* allele (Figure 7A) and this suppression was independent of AmiA, -B, or -C (Supplementary Figure 3). Furthermore, PG analysis indicated that AmiC-derived non-canonical muropeptides (TS-tetra and TS-tetra-DS-tetra) were completely absent in cells overexpressing *actS^lytM^* (Figure 7C). In addition, overexpression of *actS^lytM^* did not elicit lysis in WT cells.

Next, to examine whether any active site residues of ActS are required for the suppression of Δ*mepS* phenotype, we constructed a variant of *actS* with an Aspartate149 residue substituted by alanine (pTRC99a-*actS*^*D*149*A*^). In the LytM homolog of *Staphylococcus aureus*, the corresponding Asp214 residue is shown to be crucial for chelating a Zn^2+^ ion for its activity (Firczuk et al., 2005; Sabala et al., 2012). Similar to *actS^lytM^*, overexpression of full-length *actS* containing D149A mutation compensated the loss of *mepS*, was unable to confer cell lysis, and failed to produce AmiC-derived muropeptides (Figures 7B,C). These observations showed that Asp149 residue and the septal localization are crucial to activate AmiC but not to rescue the growth defect of Δ*mepS* mutant.

**FIGURE 8:**
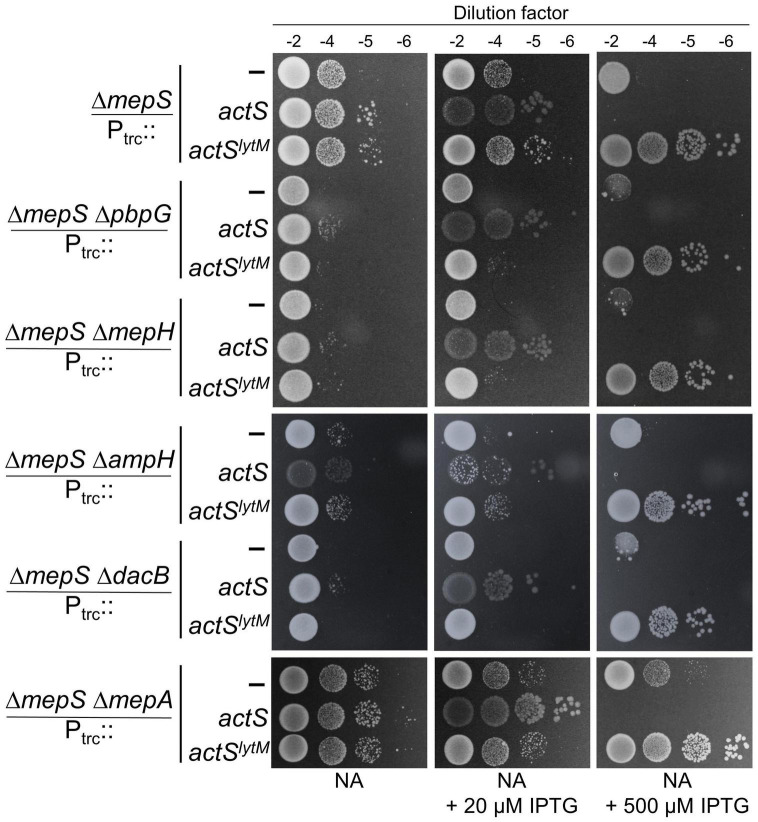
Growth advantage to Δ*mepS* mutant by ActS is not mediated by *D,D*-endopeptidases. Δ*mepS* mutant and its derivatives carrying pTRC99a vector (P_trc_::empty), vector encoding *actS* (P_trc_::*actS*), or *actS^lytM^* (P_trc_::*actS^lytM^*) were grown in LB and tested for viability on NA plates supplemented with and without IPTG at 37°C.

The above results established that in addition to activating AmiC, ActS is capable of performing an alternate function that facilitates the growth of Δ*mepS* mutant. To examine whether ActS is functioning to activate any other known PG hydrolase of *E. coli*, we introduced single-gene deletions lacking either *D,D*-endopeptidases (MepA, MepH, NlpC, YafL, AmpH, DacB, and PbpG), *L,D*-endopeptidases (MepK, LdtF), glycosylases (Slt, MltA, -B, -C, -D, -E, -F, -G, and DigH), or PG-recycling factors (AmpG, AmiD) into the Δ*mepS* mutant. However, the overexpression of *actS* or *actS^lytM^* alleviated the growth defects of all the double mutants like that of a single *mepS* deletion mutant (Figure 8 and Supplementary Figures 4–6) showing that ActS does not function *via* any of these known PG hydrolases. These observations raise the possibility of ActS performing an alternate function that is independent of cell wall hydrolysis.

## Discussion

*Escherichia coli* contains a class of LytM factors encoded by *envC*, *nlpD*, *mepM*, and *actS* (formerly, *ygeR*). Of these, EnvC and NlpD facilitate cytokinesis by activating division-specific amidases, AmiA, AmiB, and AmiC, respectively (Uehara et al., 2010). In contrast, MepM is a 4-3 cross-link specific D,D-endopeptidase involved in PG enlargement (Singh et al., 2012). In this study, we show that ActS is an activator of the amidase, AmiC. In addition, we find that ActS is able to suppress the defects of an elongation-specific endopeptidase, MepS. Interestingly, these two activities of ActS are distinctive. ActS function has been recently elaborated by two other studies, where it has been shown to activate the amidases, AmiB and AmiC (Gurnani Serrano et al., 2021; Mueller et al., 2021).

We identified *actS* as its overexpression modestly suppressed the growth defects of a mutant lacking a major elongation-specific *D,D*-endopeptidase, MepS. Extensive genetic, molecular, and microscopic analyses revealed that ActS activates the division-specific amidase, AmiC. However, interestingly, the rescue of growth defects of Δ*mepS* mutant by ActS was not mediated by AmiC, but by a yet unknown mechanism. Domain-deletion experiments confirmed the requirement of the LysM domain of ActS for activation of AmiC, but not for alleviation of growth defects of *mepS* mutant, clearly suggesting two distinct roles for ActS in PG metabolism.

### Role of ActS in Septal Peptidoglycan Hydrolysis

ActS was earlier implicated to have a minor role in cell separation as deletion of *actS* somewhat exacerbated the chaining effect caused by the absence of the LytM domain factors, EnvC, NlpD, and MepM (Uehara et al., 2009). A recent study (Mueller et al., 2021) showed that ActS preferentially stimulates AmiB and to a lesser extent AmiC in cells grown under acidic pH conditions, and an accompanying study (Gurnani Serrano et al., 2021) demonstrated that ActS activates AmiC during envelope stress. In our study, we observed that the overexpression of ActS resulted in the formation of glycan strands lacking the peptide stems (Figures 3B,C). In addition, overexpression of ActS led to rapid cell lysis in the WT cells (Figure 2D). Both these overexpression phenotypes were significantly abolished in the absence of AmiC, thereby allowing us to conclude that ActS predominantly works *via* activation of AmiC (Figures 5A,B). All these studies collectively established that similar to other LytM-domain factors, EnvC and NlpD, ActS also functions to activate a sub-set of division-specific amidases.

It is known that AmiC is recruited to the midcell during division, and NlpD stimulates AmiC by displacing its alpha-helix, exposing the occluded active site of AmiC (Bernhardt and de Boer, 2003; Peters et al., 2013). However, the mechanism of stimulation of AmiC by ActS is not yet clear. Being a paralog of NlpD, ActS may also likely operate *via* an analogous mechanism. Similar to that of EnvC and NlpD, ActS is also a late-recruiter to the cell septum as ActS-GFP localizes exclusively to the deeply constricting cells (Figure 5C; Uehara et al., 2009). The requirement of the LysM domain of ActS for the activation of AmiC (Figure 7C; Tsang et al., 2017) suggests that septal localization is a prerequisite for its function. The essentiality of the aspartate-149 residue also indicates that coordination with a zinc residue is important for the activation of AmiC (Figure 7C).

### An Alternate Role of ActS in Peptidoglycan Metabolism

The overexpression of ActS modestly rescued the growth defect of *mepS* deletion mutant on NA (Figure 2B); in addition, it weakly suppressed the growth defect of a mutant lacking both *mepS* and *mepM* indicating that ActS does not work through MepM (Figure 2C). However, *actS* deletion did not confer any discernible growth defect to Δ*mepS* or Δ*mepSM* deletion mutants under laboratory conditions. These results suggest that under normal physiological conditions, *actS* may not contribute significantly to PG expansion. Unlike ActS, overexpression of other division-specific LytM domain factors, EnvC and NlpD did not compensate for the loss of MepS, suggesting that the effect is specific only to ActS (Supplementary Figure 7). As we and others have clearly shown that ActS does not possess any biochemical activity (Gurnani Serrano et al., 2021; Mueller et al., 2021), and also that the LysM domain is not important for the rescue of MepS mutant, we presumed that overexpression of ActS may activate another PG hydrolase either directly, or indirectly *via* induction of a stress response pathway. We extensively tested the role of all known PG hydrolases in the suppression of *mepS* phenotypes by ActS overexpression (Figure 8 and Supplementary Figures 4–6); however, none of these were found to be the candidates suggesting ActS may modulate yet another effector in *E. coli*. In this context, a previous study showed that ActS-mCherry displays a patchy peripheral localization in addition to a weak septal localization in cells with a deep constriction (Uehara et al., 2009). This pattern of peripheral localization correlates well with our observations of ActS having a dual role, one to activate AmiC at the septum and an alternate activity that is independent of its septal localization. Further efforts are required to uncover the basis of suppression of Δ*mepS* growth defects by ActS.

## Data Availability Statement

The original contributions presented in this study are included in the article/Supplementary Material, further inquiries can be directed to the corresponding author.

## Author Contributions

PC, RA, and MR conceived to the study. PC, RB, and RA performed the experiments. PC and MR analyzed the data and wrote the manuscript. All authors contributed to the article and approved the submitted version.

## Conflict of Interest

The authors declare that the research was conducted in the absence of any commercial or financial relationships that could be construed as a potential conflict of interest.

## Publisher’s Note

All claims expressed in this article are solely those of the authors and do not necessarily represent those of their affiliated organizations, or those of the publisher, the editors and the reviewers. Any product that may be evaluated in this article, or claim that may be made by its manufacturer, is not guaranteed or endorsed by the publisher.
