# Supplementary material for: A LytM-Domain Factor, ActS, Functions in Two Distinctive Peptidoglycan Hydrolytic Pathways in E. coli
Source: Front Microbiol. 2022 Jun 14;13:913949. doi: 10.3389/fmicb.2022.913949 (PMC9238320; doi:10.3389/fmicb.2022.913949)

**Supplementary Information**

**A LytM-domain factor, ActS functions in two distinctive peptidoglycan hydrolytic pathways in *Escherichia coli***

Pavan Kumar Chodisetti, Raj Bahadur, R N Amrutha, Manjula Reddy*

CSIR-Centre for Cellular and Molecular Biology

Hyderabad India 500007

Running title: Role of ActS in peptidoglycan hydrolysis

Key words: Bacteria, Peptidoglycan, Cell division, YgeR, MepS, ActS, AmiC

*To whom correspondence should be addressed

Manjula Reddy

S-106, CSIR-Centre for Cellular and Molecular Biology

Habsiguda, Hyderabad, India 500007

Ph: +91-40-27192514

Fax: +91-40-27160591

E-mail: manjula@ccmb.res.in; mreddy65@gmail.com

**Supporting Materials and Methods**

**Plasmid constructions**

For PCR amplification, genomic DNA of MG1655 strain was used as a template unless otherwise indicated. Amplification of DNA was done using Phusion DNA polymerase (NEB) and clones obtained were always confirmed by DNA sequencing.

**pPK31 (pTRC99a *actS*).** The full-length *actS* gene was PCR amplified using forward and reverse primers 5’GCTCTAGATTGAGTGCGGGACGCCTG3’ and 5’AAACTGCAGtcaGCATTTTGGCTTGCTGC3’ respectively and the resulting amplified DNA fragment was cloned at XbaI-PstI sites (underlined) of pTRC99a vector to obtain pPK31. Note that the reverse primer has a stop codon (tca) indicated with lower case letters. pPK31 suppressed the growth defects of the ∆*mepS* mutant on nutrient agar at 37 °C with 10 µM IPTG.

**pPK36 (pTRC99a *actS^∆28-129^* or *actS^∆lysM^* or *actS^lytM^*).** Throughout the manuscript, this plasmid construct is referred to as pTRC99a *actS^lytM^*. pPK36 is a truncated version of ActS where the LysM domain is removed and the LytM domain fused to its native signal peptide. For this purpose, two overhang primers were synthesized, and a 3 step PCR was performed. The overhangs of forward and reverse primers have complementarity with a signal peptide and LytM respectively. In the first PCR, an overhang forward primer 5’CTGCTTTTGGCGGGCTGTTCGACGACAGGGAAAGTTATCATGCCG3’ and a common reverse primer 5’AAACTGCAGtcaGCATTTTGGCTTGCTGC3’ were used to amplify the LytM domain of ActS where its N-terminal region is complementary to the signal peptide. Then in the second PCR, a common forward primer 5’GCTCTAGATTGAGTGCGGGACGCCTG3’ and an overhang reverse primer 5’GCATGATAACTTTCCCTGTCGTCGAACAGCCCGCCAAAAGCAGTCC3’ were used to amplify native signal peptide in which its C-terminal region is complementary to the LytM domain. Common reverse primer has a stop codon (tca) indicated with lower case letters whereas restriction sites are underlined. In the third PCR step, first and second PCR products were mixed in a 1:1 molar ratio and end filling was done using common forward and reverse primers to obtain a fusion where the signal peptide is fused to the LytM domain without the LysM domain. The final PCR product was digested with XbaI and PstI restriction enzymes and cloned into the pTRC99a vector to obtain the pPK36 plasmid.

**pPK37 (pTRC99a *actS^D149A^*).** For the generation of a site-directed variant of *actS*, D149A, a 3 step PCR was performed. In the first PCR step, the N-terminal fragment of the *actS* gene was amplified using a common forward primer 5’GCTCTAGATTGAGTGCGGGACGCCTG3’and a reverse primer containing the desired mismatch 5’GCGGCAATAAAGGGATTG**CG**ATCTCAGCTCCACGGGGTAC3’. In the second PCR step, the C-terminal fragment of the *actS* gene was amplified using a forward primer containing the desired mismatch 5’GTACCCCGTGGAGCTGAGAT**CG**CAATCCCTTTATTGCCGC3’ and a common reverse primer 5’AAACTGCAGtcaGCATTTTGGCTTGCTGC3’. Desired mismatch (in bold and underlined) codes for an alanine instead of aspartic acid at position 149 in ActS. Reverse primer has a stop codon (tca) indicated with lower case letters. Then, both the PCR products were mixed in a 1:1 molar ratio, and end filling was done by a final PCR step using common forward and reverse primer. The obtained PCR product was digested with XbaI-PstI (underlined) and cloned into a pTRC99a vector to obtain pPK37 which was further confirmed for the presence of D149A mutation by DNA sequencing.

**pPK33 (pBAD18 *actS*-sfGFP).** A fragment that encodes *actS*^1-251^ along with its native RBS was cloned into a pBAD18-sfGFP vector using a forward primer 5’CTAGCTAGCCGAGGAAAGATTTTGAGTGCG3’ and a reverse primer 5’CCCCCCGGGTGCATTTTGGCTTGCTGCC3’ respectively in between NheI and XmaI restriction sites (underlined) to generate ActS with a C-terminal sfGFP fusion.

**pPK34 (pET21b *actS*^27-251^ or *actS^fl^*).** A fragment that encodes *actS*^27-251^ was cloned into a pET21b vector in between NdeI and XhoI sites using a forward primer 5’CGCCATATGTCGGGTAGCAAATCATCC3’ and a reverse primer 5’CCGCCTCGAGGCATTTTGGCTTGCTGCC3’ to generate ActS^FL^ with a C-terminal 6XHis fusion.

**pPK35 (pET21b *actS*^130-251^ or *actS^lytM^*).** A fragment that encodes *actS*^130-251^ was cloned into the pET21b vector in between NdeI and XhoI sites using a forward primer 5’ GGAATTCCATATGACGACAGGGAAAGTTATCATG3’ and a reverse primer 5’CCGCCTCGAGGCATTTTGGCTTGCTGCC3’ to generate ActS^LytM^ with C-terminal 6XHis fusion.

**pPK32 (pET21b *cwlO*^340-473^).** A fragment encoding *cwlO*^340-473^ was PCR amplified using *Bacillus subtilis* 168 genomic DNA as the template with the forward and reverse primers 5’GGAATTCCATATGTCTGGCGGAATTGAAGGCGC3’ 5’CCGCTCGAGTTGAACAACACGTCTTAC3’ and cloned into pET21b vector in between NdeI and XhoI sites to generate CwlO^340-473^ with C-terminal 6XHis fusion.

**pPK38 (pTRC99a *actS^1-251^*-*his*).**

pPK38 was constructed similar to that of pPK31, except that the reverse primer (with HindIII restriction site) used here was 5’CCCAAGCTTTCAatggtgatggtgatggtgGCATTTTGGCTTGCTGCCC3’. Codons encoding 6X His tag are indicated in lower case letters.

**pPK39 (pTRC99a *actS^D149A^*-*his*)**.

pPK39 was constructed similar to pPK37, except that the common reverse primer (with HindIII restriction site) used here was 5’CCCAAGCTTTCAatggtgatggtgatggtgGCATTTTGGCTTGCTGCCC3’. Codons encoding 6X His tag is indicated in lower case letters.

**Figure Legends:**

**Figure S1.** ActS activates AmiC**. (A)** WT, Δ*amiA*, Δ*amiB*, or Δ*amiC* mutants carrying pTRC99a vector alone (P_trc_::empty) or vector encoding *actS* (P_trc_::*actS*) were grown in LB and tested for viability on LB plates supplemented with 0 and 100 µM IPTG. **(B)** WT, Δ*amiAB*, Δ*amiBC*, or Δ*amiAC* mutants carrying vector (P_trc_::empty) or vector encoding *actS* (P_trc_::*actS*) were subjected to microscopy as described in Materials and Methods. Strains were grown overnight and diluted 1:100 into fresh LB broth supplemented with 50 µM IPTG and grown at 37°C till OD_600_ of 0.5. Cells were collected and visualized with DIC optics. Arrows indicate cell lysis, and the scale bar represents 5 μm.

**Figure S2.** Overproduction of amidases does not suppress the ∆*mepS* phenotype. ∆*mepS* mutant carrying vector (P_T5-lac_::empty), vector encoding *amiA* (P_T5-lac_::*amiA*), *amiB* (P_T5-lac_::*amiB*), or *amiC* (P_T5-lac_::*amiC*) were grown in LB and tested for viability on NA plates supplemented with indicated IPTG at 37°C.

**Figure S3.** ActS alleviates the growth defects of ∆*mepS* not via AmiC. Indicated strains carrying the empty vector (P_trc_::empty) and vector encoding *actS^lytM^* (P_trc_::*actS^lytM^*) were grown overnight in LB broth and subjected to viability assay on NA supplemented with 0 and 500 µM IPTG.

**Figure S4.** ActS confers a growth advantage to ∆*mepS* mutant not via L,D-endopeptidases. Δ*mepS* mutant and its derivatives carrying pTRC99a vector (P_trc_::empty), vector encoding *actS* (P_trc_::*actS*), or *actS^lytM^* (P_trc_::*actS^lytM^*) were grown in LB and tested for viability on NA plates supplemented with indicated IPTG at 37°C.

**Figure S5.** ActS confers a growth advantage to ∆*mepS* mutant not via lytic transglycosylases. Δ*mepS* mutant and its derivatives carrying pTRC99a vector (P_trc_::empty), vector encoding *actS* (P_trc_::*actS*), or *actS^lytM^* (P_trc_::*actS^lytM^*) were grown in LB and tested for viability on NA plates supplemented with indicated IPTG at 37°C.

**Figure S6.** ActS confers a growth advantage to ∆*mepS* mutant not via PG-recycling factors (AmpG and AmiD) and other factors (NlpC, YafL, and DigH). Δ*mepS* mutant and its derivatives carrying pTRC99a vector (P_trc_::empty), vector encoding *actS* (P_trc_::*actS*), or *actS^lytM^* (P_trc_::*actS^lytM^*) were grown in LB and tested for viability on NA plates supplemented with indicated IPTG at 37°C.

**Figure S7.** Viability assay to test the suppression of growth defects of ∆*mepS* by LytM-domain proteins. Δ*mepS* mutant carrying empty vector (P_T5-lac_::), vector encoding *envC* (P_T5-lac_::*envC*), *nlpD* (P_T5-lac_::*nlpD*), *mepM* (P_T5-lac_::*mepM*), or *actS* (P_T5-lac_::*actS*) were grown in LB and tested for viability on NA plates supplemented with IPTG at 37°C.

**Table S1. Muropeptide composition of various strains**

|  | % Area of muropeptide peaks^a^ (P_trc_::*actS*) | | | | | | | |  |
| --- | --- | --- | --- | --- | --- | --- | --- | --- | --- |
| Muropeptide (Peak) | WT | ∆*amiA* | ∆*amiB* | | ∆*amiC* | | | ∆*amiABC* | |
| Tri (1) | 5.2 | 7.6 | | 5 | | 8 | 6.9 | |  |
| Tetra (2) | 36.3 | 33.9 | | 38 | | 36.7 | 36.2 | |  |
| Tri-Lys-Arg | 3.3 | 3.2 | | 3.2 | | 2.3 | 2.6 | |  |
| **TS-tetra (A)** | **1.5** | **1.2** | | **1.3** | | **0.2** | **0.14** | | |
| Tetra−tetra (5) | 29.6 | 29 | | 29 | | 30 | 28.5 | | |
| **TS-tetra−DS-tetra (B)** | **3.7** | **3** | | **4.1** | | **1.4** | **0.3** | | |
|  |  |  | |  | |  |  | | |

^a^Muropeptide analysis was done by calculating the relative percentage area of each muropeptide from the HPLC chromatograms. Peak A and B muropeptide values are shown in bold.

**Table S2. Strains used in this study**

Strains Genotype^a^ Source/Reference

BW25113 *lacI*^q^ *rrnB*3 Δ*lacZ*4787 Δ(*araBAD*)567 Baba *et al*., 2006

Δ(*rhaBAD*)568 *hsdR*514

BL21 (λDE3) *ompT* rB^−^ mB^−^ (P_lac_*UV5*::T7*gene*1) Lab collection

DH5α *F– hsdR17 deoR recA1 endA1phoA supE44 thi-1*

*gyrA96 relA1*Δ*(lacZYA-argF)U169* φ*80dlacZ* ΔM15 Lab collection

MG1655 *rph*1 *ilvG rfb*-50 Lab collection

MGNA-A001 *Bacillus subtilis 168* NBRP

JW2833 BW25113 ∆*actS*(*ygeR*)::Kan Baba *et al*., 2006

JW2428 BW25113 ∆*amiA*::Kan Baba *et al*., 2006

JW4127 BW25113 ∆*amiB*::Kan Baba *et al*., 2006

JW5449 BW25113 ∆*amiC*::Kan Baba *et al*., 2006

JW0214 BW25113 ∆*ldtF*(*yafK*)::Kan Baba *et al*., 2006

JW5355 BW25113 ∆*pbpG*::Kan Baba *et al*., 2006

JW5270 BW25113 ∆*mepH*::Kan Baba *et al*., 2006

JW5052 BW25113 ∆*ampH*::Kan Baba *et al*., 2006

JW3149 BW25113 ∆*dacB*::Kan Baba *et al*., 2006

JW2325 BW25113 ∆*mepA*::Kan Baba *et al*., 2006

JW2784 BW25113 ∆*mltA*::Kan Baba *et al*., 2006

JW2671 BW25113 ∆*mltB*::Kan Baba *et al*., 2006

JW5481 BW25113 ∆*mltC*::Kan Baba *et al*., 2006

JW5018 BW25113 ∆*mltD*::Kan Baba *et al*., 2006

JW5821 BW25113 ∆*mltE*::Kan Baba *et al*., 2006

JW2542 BW25113 ∆*mltF*::Kan Baba *et al*., 2006

JW1083 BW25113 ∆*mltG*::Kan Baba *et al*., 2006

JW1486 BW25113 ∆*digH*::Kan Baba *et al*., 2006

JW1698 BW25113 ∆*nlpC*::Kan Baba *et al*., 2006

JW0217 BW25113 ∆*yafL*::Kan Baba *et al*., 2006

JW0423 BW25113 ∆*ampG*::Kan Baba *et al*., 2006

JW0851 BW25113 ∆*amiD*::Kan Baba *et al*., 2006

MR810 MG1655 Δ*mepS*::*frt* Singh *et al*., 2015

PC301 MG1655 ∆*actS*::*frt* This study

PC305 MG1655 ∆*amiA*::*frt* This study

PC306 MG1655 ∆*amiB*::*frt* This study

PC307 MG1655 ∆*amiC*::*frt* This study

PC311 PC305 ∆*amiB*::*frt* This study

PC312 PC306 ∆*amiC*::*frt* This study

PC313 PC305 ∆*amiC*::*frt* This study

PC315 PC311 ∆*amiC*::*frt* This study

PC316 MR810 ∆*ldtF*::Kan This study

PC317 MR810 ∆*pbpG*::Kan This study

PC318 MR810 ∆*mepH*::Kan This study

PC319 MR810 ∆*ampH*::Kan This study

PC320 MR810 ∆*dacB*::Kan This study

PC321 MR810 ∆*mepA*::Kan This study

PC322 MR810 ∆*mltA*::Kan This study

PC323 MR810 ∆*mltB*::Kan This study

PC324 MR810 ∆*mltC*::Kan This study

PC325 MR810 ∆*mltD*::Kan This study

PC326 MR810 ∆*mltE*::Kan This study

PC327 MR810 ∆*mltF*::Kan This study

PC328 MR810 ∆*mltG*::Kan This study

PC329 MR810 ∆*digH*::Kan This study

PC330 MR810 ∆*nlpC*::Kan This study

PC331 MR810 ∆*yafL*::Kan This study

PC332 MR810 ∆*ampG*::Kan This study

PC333 MR810 ∆*amiD*::Kan This study

^a^Deletion alleles used in this study are sourced from Keio collection (Baba *et al*., 2006). The deletion mutations were used after testing for their authenticity (by linkage analysis, PCR and sequence analysis) and introduced into different strain backgrounds by P1 phage-mediated transduction [Miller JH (1992)].

**Table S3. Plasmids used in this study**

Plasmid Relevant Features Source/Reference

pCA24N Cm^R^, *lacI^q^,* P_T5-lac_:: [Kitagawa, *et al*., 2005]

pCA24N-*actS* Cm^R^, *lacI^q^,* P_T5-lac_::*actS* [Kitagawa, *et al*., 2005]

pCA24N-*amiA* Cm^R^, *lacI^q^,* P_T5-lac_:: *amiA* [Kitagawa, *et al*., 2005]

pCA24N-*amiB* Cm^R^, *lacI^q^,* P_T5-lac_:: *amiB* [Kitagawa, *et al*., 2005]

pCA24N-*amiC* Cm^R^, *lacI^q^,* P_T5-lac_:: *amiC* [Kitagawa, *et al*., 2005]

pCP20 pSC101(Ts), Amp^R^, Cm^R^, Flp [Datsenko & Wanner 2000]

pET21b ColE1, Amp^R^, *lacI*q, T7*lac* Novagen

pMN83 pBAD33 *mepS*¸ Cm^R^ [Singh *et al*., 2012]

pTRC99a ColE1, Amp^R^, *lacI*q, P_trc_ Lab collection

pPK31 pTRC99a-*actS* (*actS*^1-251^) This study

pPK32 pET21b *cwlO*^340-473^  This study

pPK33 pBAD18 *actS*-*sfGFP* This study

pPK34 pET21b *actS*^27-251^ (*actS^fl^*) This study

pPK35 pET21b *actS*^130-251^ (*actS^lytM^*) This study

pPK36 pTRC99a *actS^lytM^* (*actS^∆28-129^* or *actS^∆lysM^*) This study

pPK37 pTRC99a *actS^D149A^* (*actS^D149A^*) This study

pPK38 pTRC99a *actS*^1-251^-his This study

pPK39 pTRC99a *actS*^D149A^-his This study

**References**

Baba, T., Ara, T., Hasegawa, M., Takai, Y., Okumura, Y., Baba, M., *et al*. (2006). Construction of *Escherichia coli* K-12 in-frame, single-gene knockout mutants: the Keio collection. *Mol. Syst. Biol*. 2:2006.0008. doi: 10.1038/msb4100050

Datsenko, K., A., and Wanner, B., L. (2000) One-step inactivation of chromosomal genes in *Escherichia coli* K-12 using PCR products. *Proc. Natl. Acad. Sci. U. S. A*. 97, 6640–6645. doi: 10.1073/pnas.120163297

Kitagawa, M., Ara, T., Arifuzzaman, M., Ioka-Nakamichi, T., Inamoto, E., Toyonaga, H., *et al.* (2005). Complete set of ORF clones of *Escherichia coli* ASKA library (a complete set of *E. coli* K-12 ORF archive): unique resources for biological research. *DNA Res*. 12, 291-299. doi: 10.1093/dnares/dsi012

Miller JH (1992) A Short Course in Bacterial Genetics: A Laboratory Manual and Handbook for *Escherichia coli* and Related Bacteria (Cold Spring Harbor Laboratory, Cold Spring Harbor, NY).

Singh SK, Saisree L, Amrutha RN, and Reddy M (2012) Three redundant murein endopeptidases catalyse an essential cleavage step in peptidoglycan synthesis of *Escherichia coli* K12. *Mol Microbiol.* 86, 1036–1051. doi: 10.1111/mmi.12058

Singh, S. K., Parveen, S., SaiSree, L., and Reddy, M. (2015) Regulated proteolysis of a cross-link–specific peptidoglycan hydrolase contributes to bacterial morphogenesis. *Proc. Natl. Acad. Sci. U. S. A*. 112, 10956–10961. doi: 10.1073/pnas.1507760112


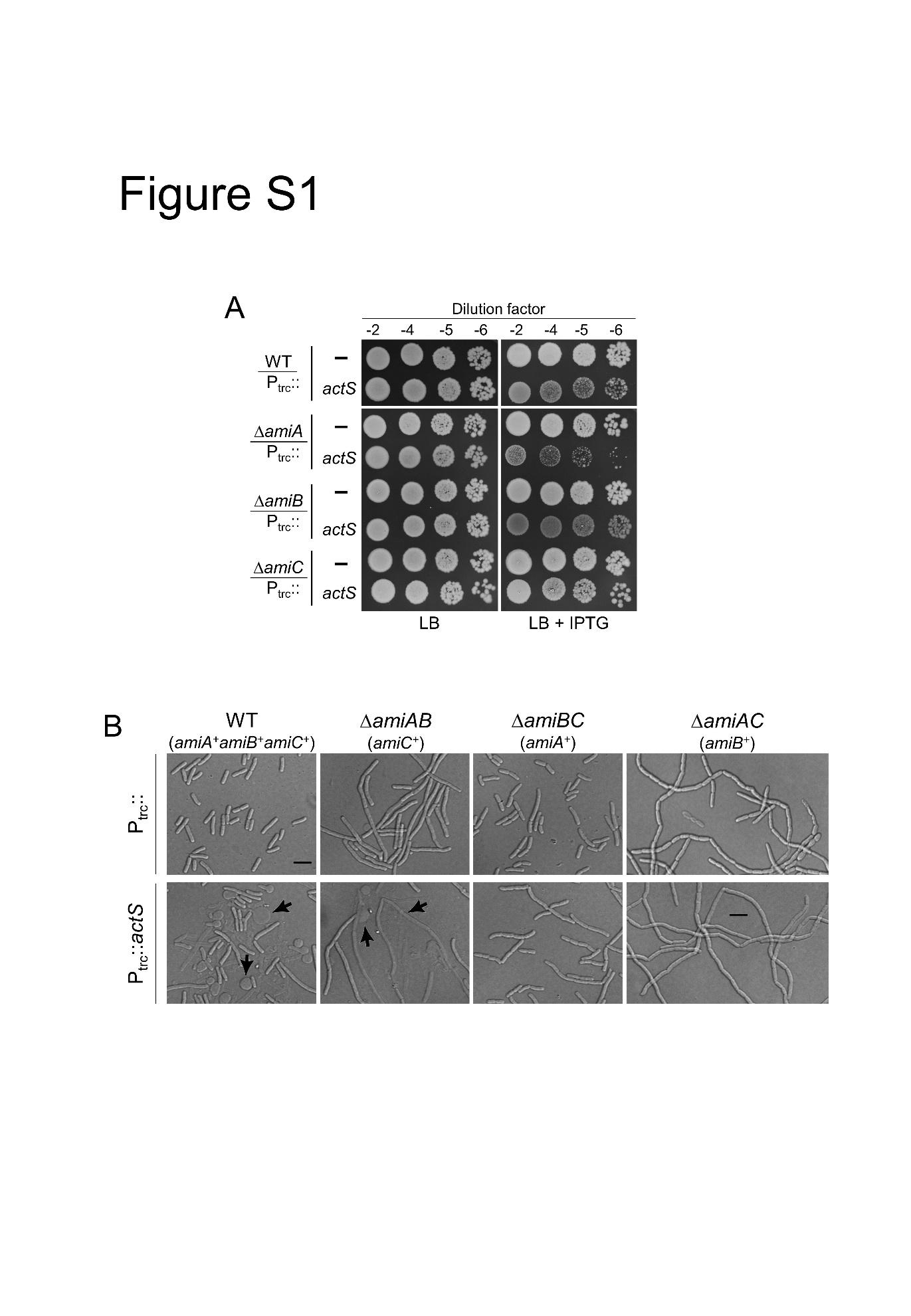

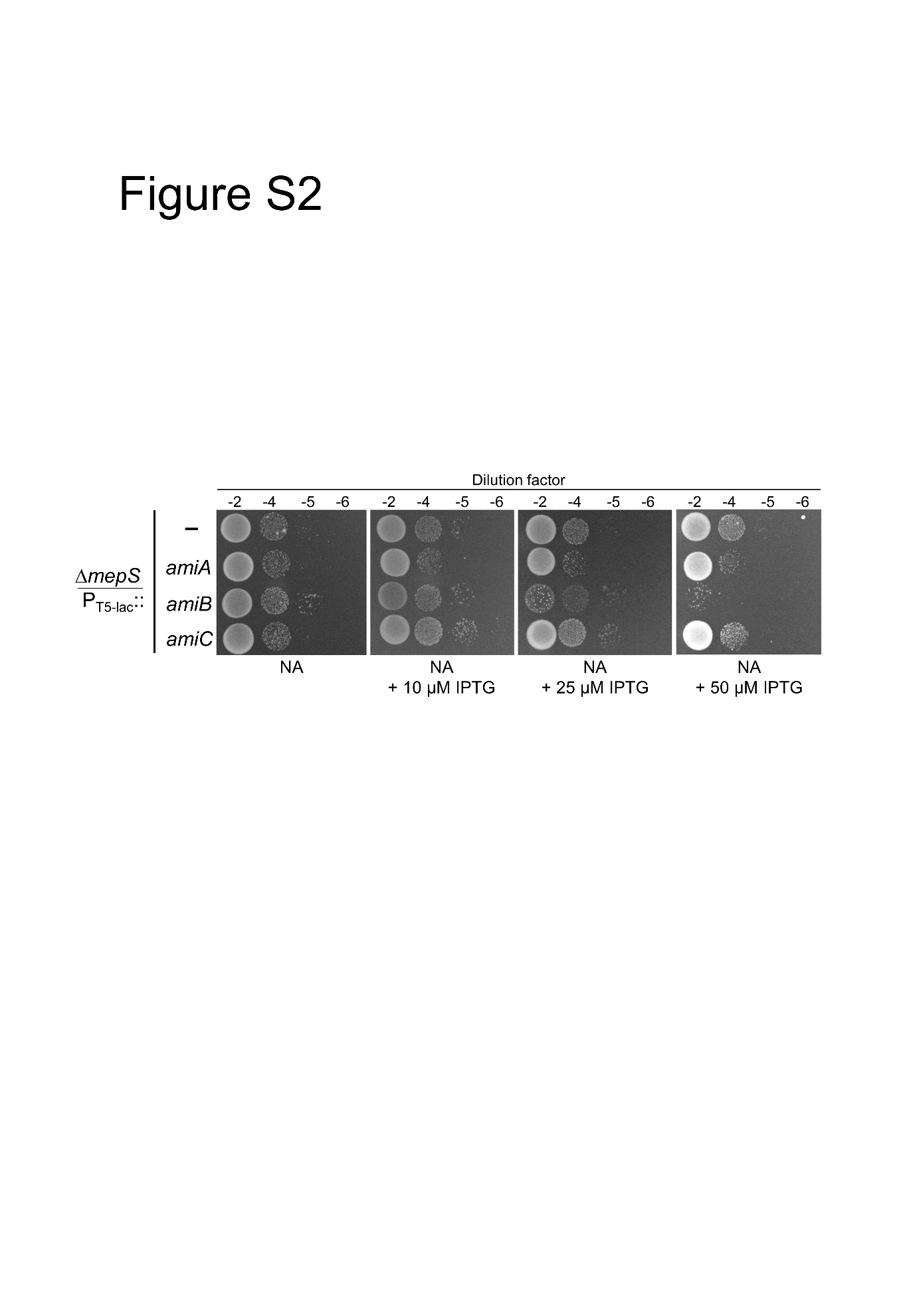


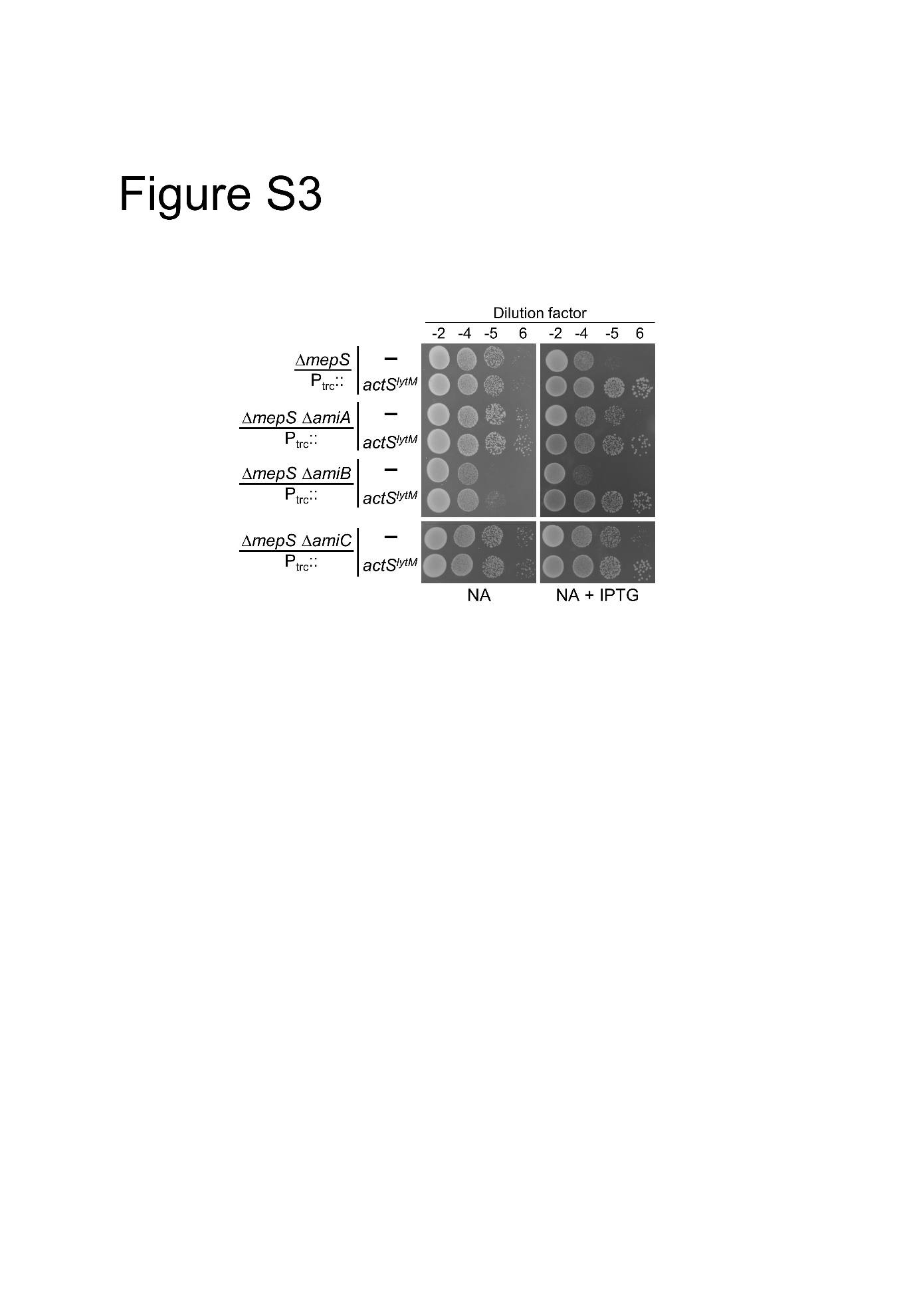


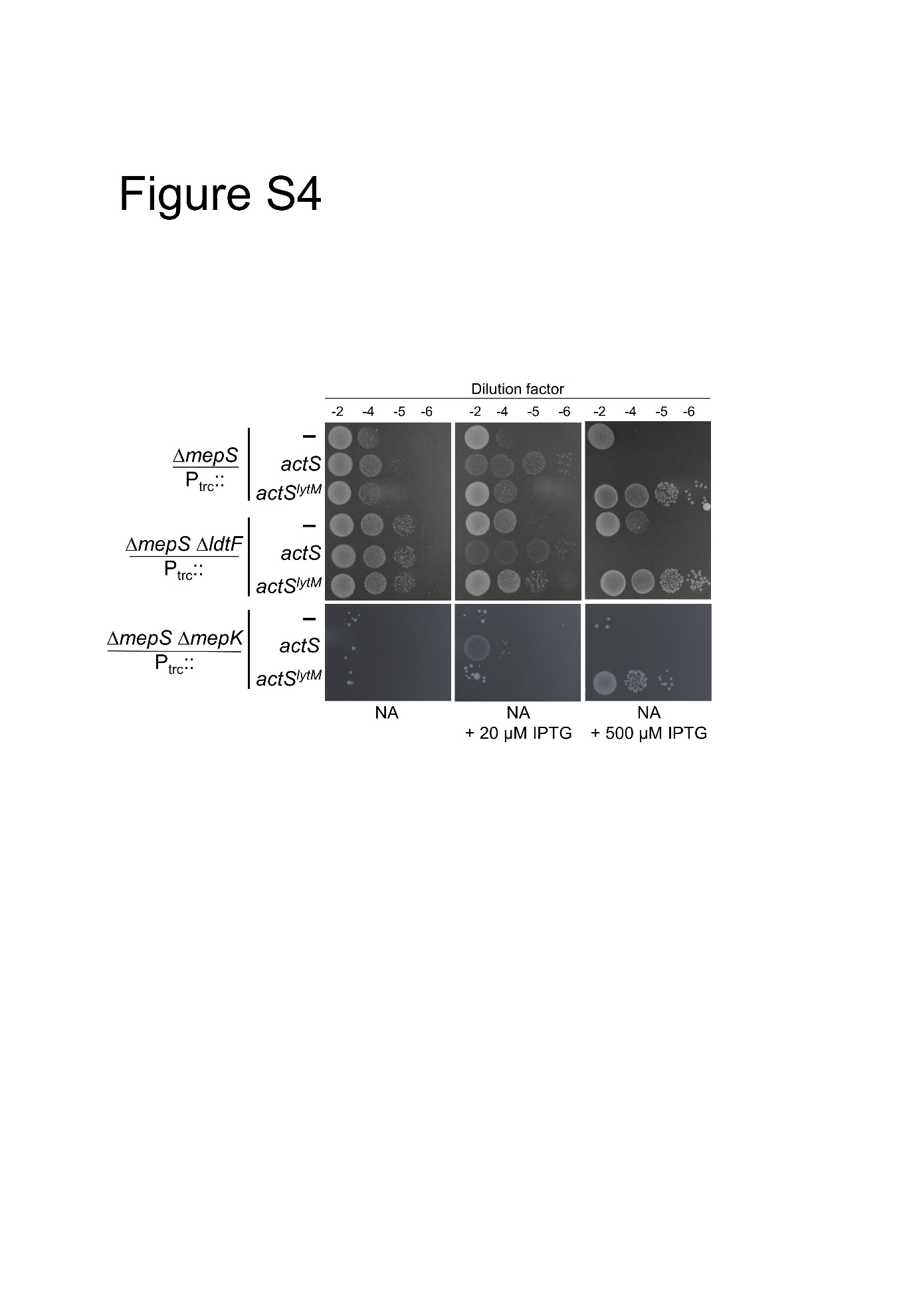


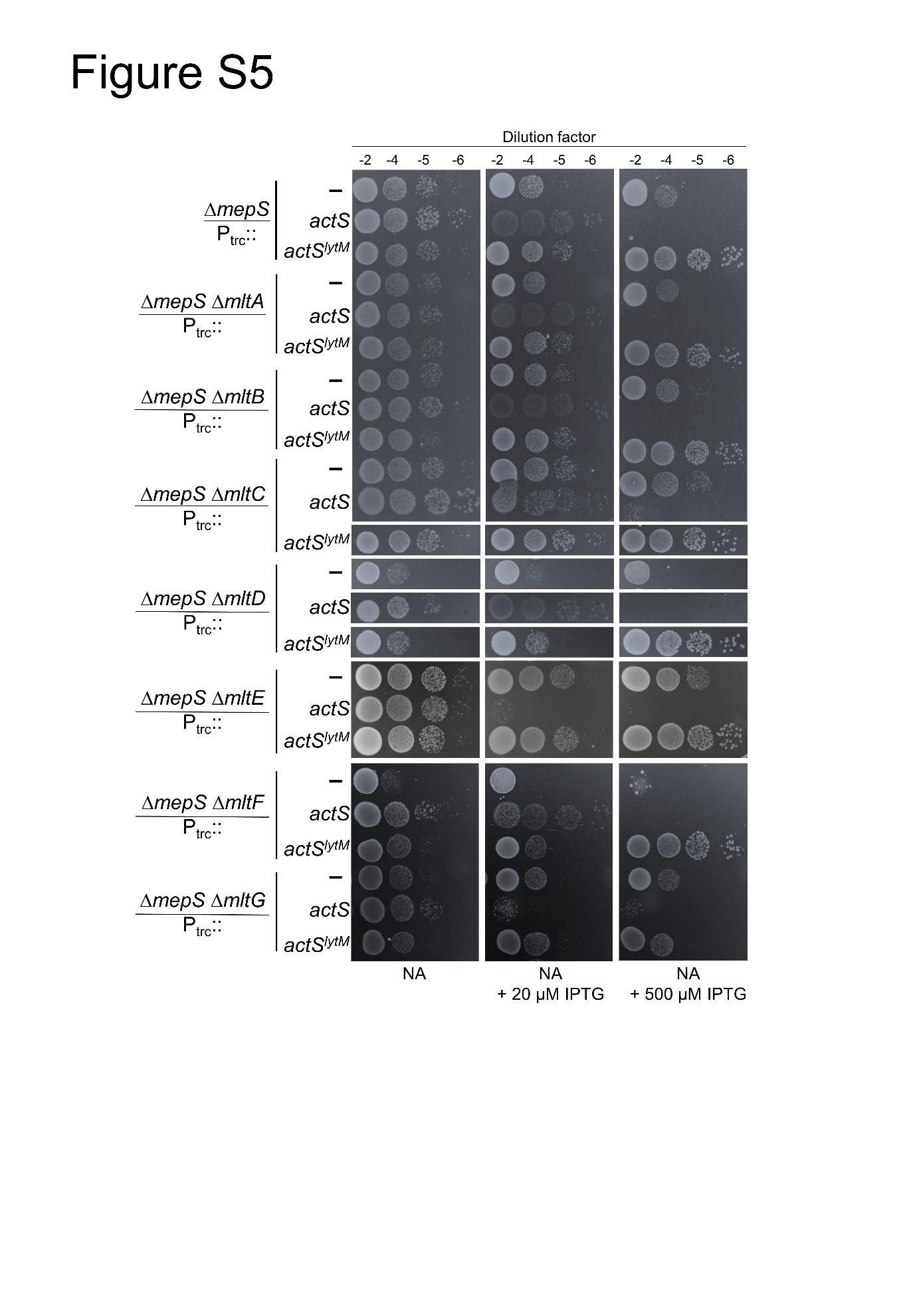


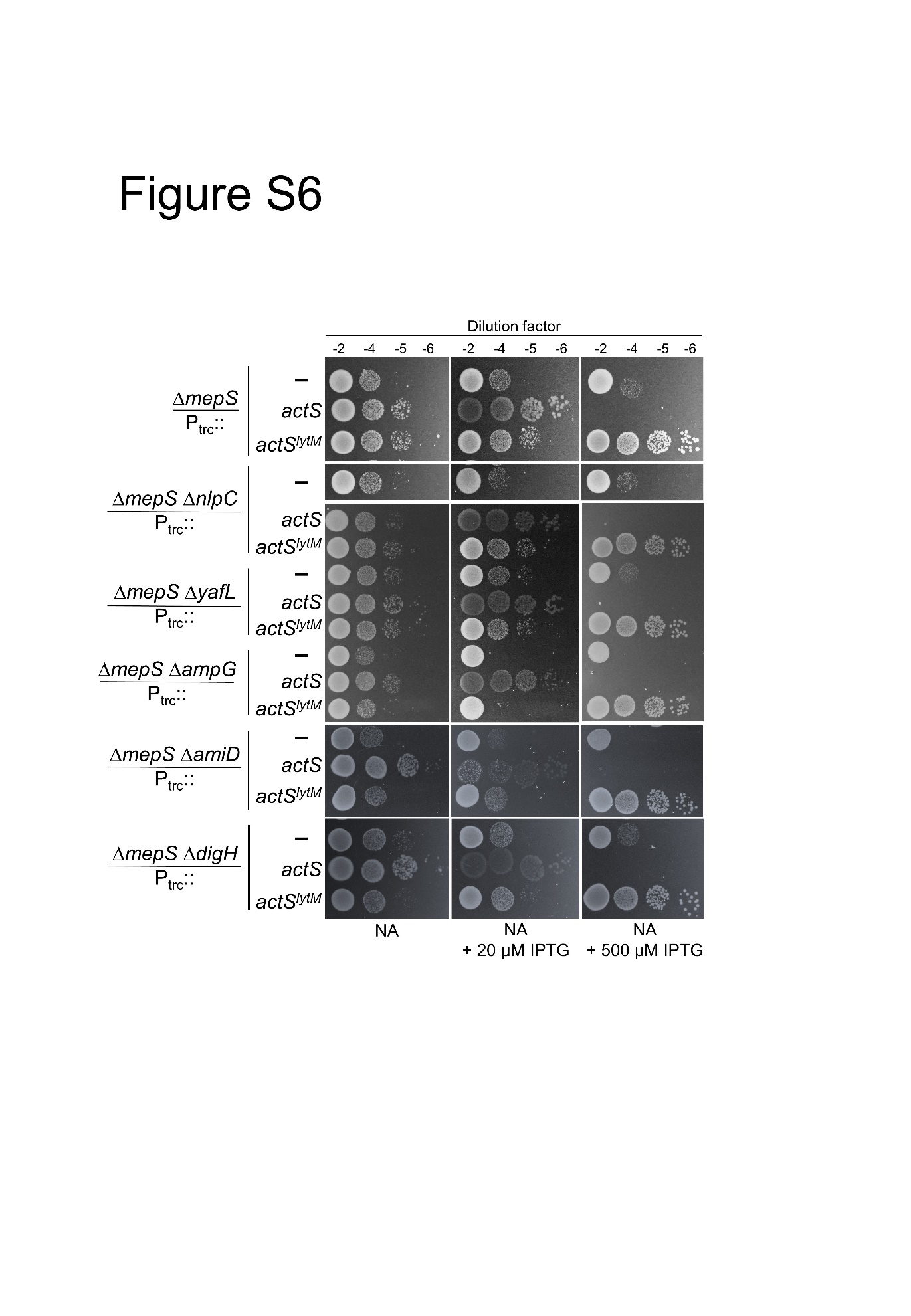


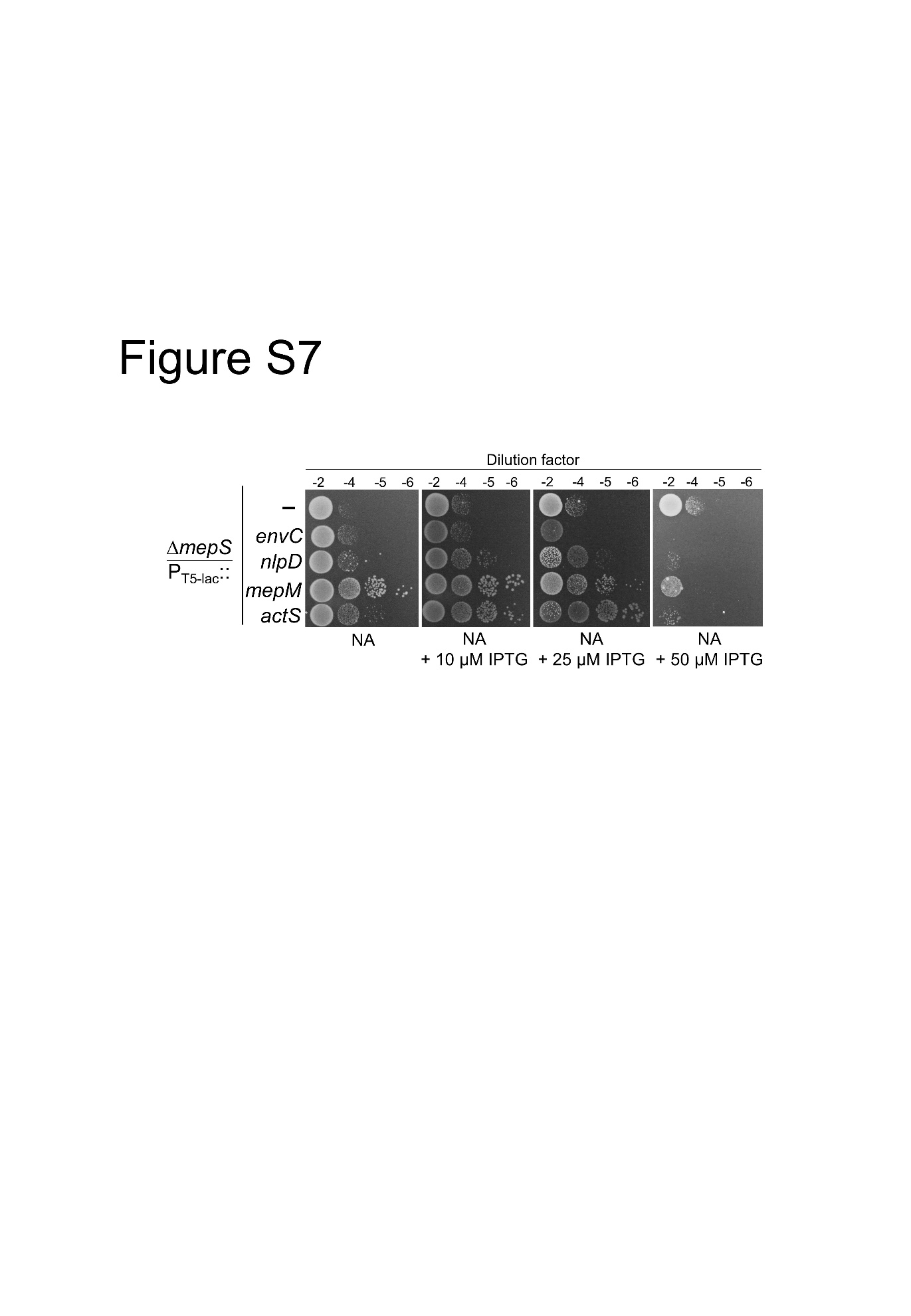

Supplement: Supplementary file 1 [file Data_Sheet_1.docx]
